# Organ–Organ Crosstalk and Alcoholic Liver Disease

**DOI:** 10.3390/biom7030062

**Published:** 2017-08-16

**Authors:** Lauren G. Poole, Christine E. Dolin, Gavin E. Arteel

**Affiliations:** Department of Pharmacology and Toxicology, University of Louisville Health Sciences Center, Louisville, KY 40292, USA; lgpool01@louisville.edu (L.G.P.); christine.dolin@louisville.edu (C.E.D.)

**Keywords:** ethanol, hepatic, pulmonary, inflammation, organ–organ axes

## Abstract

Alcohol consumption is a common custom worldwide, and the toxic effects of alcohol on several target organs are well-understood. Given the poor prognosis of treating clinically-relevant alcoholic liver disease (ALD) (i.e., alcoholic hepatitis (AH) and cirrhosis), additional research is required to develop more effective therapies. While the stages of ALD have been well-characterized, targeted therapies to prevent or reverse this process in humans are still needed. Better understanding of risk factors and mechanisms underlying disease progression can lead to the development of rational therapies to prevent or reverse ALD in the clinic. A potential area of targeted therapy for ALD may be organ–organ communication in the early stages of the disease. In contrast to AH and end-stage liver diseases, the involvement of multiple organs in the development of ALD is less understood. The impact of these changes on pathology to the liver and other organs may not only influence disease progression during the development of the disease, but also outcomes of end stages diseases. The purpose of this review is to summarize the established and proposed communication between the liver and other organ systems that may contribute to the development and progression of liver disease, as well as to other organs. Potential mechanisms of this organ–organ communication are also discussed.

## 1. Alcohol Use and Its Impact

**Figure 1:**
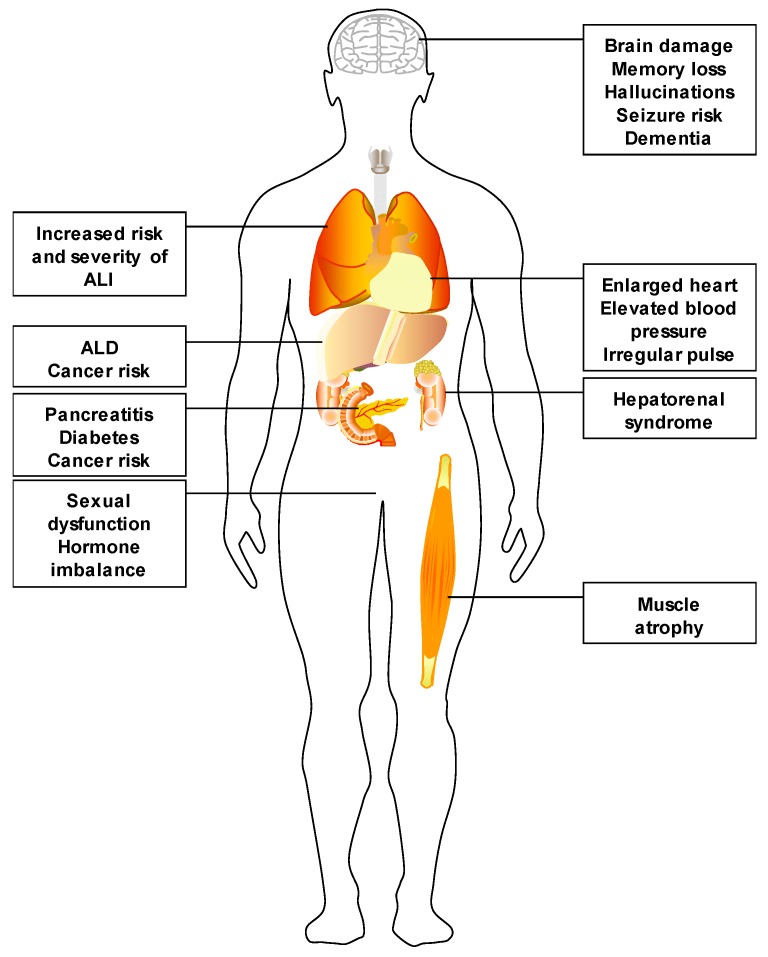
Organs targeted by alcohol abuse. Alcohol abuse is known to injury several organs in the human body, including liver, brain and pancreas. Furthermore, alcohol abuse is associated with dysfunction/injury in several other organs, such as the lungs, muscles and respiratory tract. It is likely that the metabolic demand of metabolizing pharmacologically-relevant (mM range) concentrations of alcohol explains, at least in part, the broad impact of this toxin. ALD: alcoholic liver disease; ALI: acute lung injury.

According to the National Survey on Drug Use and Health conducted in 2015, 86.4% of U.S. adults report consuming alcohol at some point in their lives [1]. Additionally, 15.1 million adults reported having an alcohol use disorder [1]. Alcohol abuse has been linked to a number of detrimental health effects. In fact, alcohol is known to contribute to roughly 200 disease states [2]. Chronic alcohol consumption/abuse directly damages several organs, including the liver [3], lung [4], skeletal muscle and heart [5], the brain [6], and the pancreas [7] (See Figure 1). Furthermore, alcohol is considered a group 1 carcinogen for cancers of the gastrointestinal (GI) tract, liver, breast and pancreas by the International Agency for Research on Cancer [8]. Ultimately, alcohol consumption is responsible for ~6% of all disability-adjusted life years (DALYs) lost in the United States [9], most of which are attributable to alcohol-induced toxicity as opposed to alcohol-related accidents.

## 2. Alcoholic Liver Disease

The liver is strategically located between the intestinal tract and the rest of the body, making it a critical organ in the clearance of toxins and xenobiotics, including alcohol, that enter the portal blood. The concentration of alcohol found in the portal blood is at least 2–3-fold higher than that in the systemic circulation [10]. Additionally, the liver is the primary site of alcohol metabolism, which produces toxic intermediate metabolites (e.g., acetaldehyde). Therefore, it is not surprising that the liver is a primary target of alcohol toxicity.

Alcoholic liver disease (ALD) is a global health burden, impacting millions of patients each year. ALD is actually a well-characterized spectrum of disease states, ranging from simple steatosis (i.e., fatty liver), to steatohepatitis (characterized by inflammation and necrosis), and, ultimately, to fibrosis and cirrhosis. Alcohol consumption increases the risk of developing ALD in a dose and time-dependent manner [11,12]; however, only a small fraction of even the heaviest drinkers will develop the most serious stages of the disease. This finding suggests that other factors (e.g., environmental or genetic) also contribute to overall risk [13]. Key aspects of currently-available ALD therapies focus on maintaining abstinence in the alcoholic and treating sequelae associated with severe disease, such as acute alcoholic hepatitis or cirrhosis [14]. Without a successful liver transplant, the effects of decompensation (e.g., hepatorenal syndrome) usually lead to the death of the patient. [15]. Additionally, preexisting cirrhosis increases the overall risk for hepatocellular carcinoma (HCC) by roughly 20-fold, even in the case of compensated cirrhosis [16]; this cancer has a dismal prognosis, with five year survival almost nil [17].

End-stage decompensated liver disease and alcoholic hepatitis (AH) are widely recognized as systemic disorders. In fact, in patients with these sequelae of ALD, cause of death is usually due to multiple organ failure, rather than liver injury, per se [18]. For example, over 30% of AH patients developed multiple organ failure, including renal, circulatory, respiratory, muscle loss and neurological complications [19]. Furthermore, patients with AH are also susceptible to the development of systemic inflammatory response syndrome (SIRS; [19]), which has also been associated with increased mortality. Taken together, it is universally understood that the clinical sequelae of AH and cirrhosis involve multiple organs.

Given the poor prognosis of treating clinically-relevant ALD (i.e., AH and cirrhosis), additional research is needed to develop more effective therapies. While the stages of ALD have been well-characterized, targeted therapies to prevent or reverse this process in humans are still needed. Better understanding of risk factors and mechanisms underlying disease progression can lead to the development of rational therapies to prevent or reverse ALD in the clinic. A potential area of targeted therapy for ALD may be organ–organ communication in the early stages of the disease. In contrast to AH and end-stage liver diseases, the involvement of multiple organs in the development of ALD is less understood. The impact of these changes on pathology in the liver and other organs may not only influence disease progression during the development of the disease, but also the outcomes of end stages diseases. The purpose of this review is to summarize the established and proposed communication between the liver and other organ systems that may contribute to the development and progression of liver disease, as well as to other organs. Potential mechanisms of this organ–organ communication are also discussed.

## 3. Mechanisms of Inter-Organ Communication

There are many mechanisms by which cells in one organ can communicate at long distances with the cells of another tissue. This review will highlight the roles of nutrients, hormones and hepatokines, innervation, extracellular vesicles, cytokines and chemokines, and molecular “danger signals” such as pathogen-associated molecular patterns (PAMPS) and danger-associated molecular patterns (DAMPS).

### 3.1. Nutrients, Hormones and Hepatokines

In addition to its central role in xenobiotic metabolism, the liver is critical for maintaining metabolic homeostasis and for the synthesis and processing of lipids and carbohydrates [20,21]. It is also the major site of synthesis of several key proteins and bile acids that are critical for the normal uptake of vitamins and lipids. The liver plays an absolutely critical role in supplying fuel to other organs (e.g., muscle, heart and brain), especially during the postabsorptive state. Therefore, alterations in the flux of carbohydrates and lipids through the liver can indirectly impact distal organs, as their energy sensing mechanisms respond to these changes. Nutrient levels in the liver also directly mediate responsive changes in the central nervous system (CNS) via glucose sensing afferent neurons in the liver and other organs; these sensors are hypothesized to mediate rapid central responses to short-term energy status alterations.

There is intricate crosstalk between these metabolic systems, which is controlled by a complex interplay of nuclear receptors, intracellular signaling pathways and transcription factors. Hormones and other endocrine mediators play a key role in regulating these responses. The liver is well known to respond to several endocrine hormones, including insulin, glucagon, thyroid hormones and cortisol. The liver also produces several hormones that can mediate several extrahepatic effects, such as insulin-like growth factor, angiotensinogen and thrombopoietin. Furthermore, it has become increasingly clear that the liver produces several endocrine-like “hepatokines” that play key roles in regulating extrahepatic responses (e.g., fibroblast growth factor 21 (FGF21); [22,23]). The net effect of these interactions is to generate an organ that rapidly responds to endocrine signals, as well as rapidly produces endocrine signals in response to stimuli.

### 3.2. Innervation

As mentioned above, the liver has several afferent neurons that mediate and coordinate its responses with extrahepatic targets, especially the CNS. This circuitry plays a key role in metabolic homeostasis, stress responses, and inflammation [24,25,26,27]. Experimental evidence suggests that the sympathetic nervous system and ethanol interact with and partially mimic each other. Indeed, acute alcohol intoxication increases the levels of adrenaline and noradrenaline in the plasma [28]. Furthermore, blocking this interaction via adrenalectomy, hepatic vagotomy, or adrenorecepor blockade attenuates the alcohol-induced hypermetabolic state in the liver [29,30]. Additionally, repeated ethanol administration increases the gene expression of enzymes required for catecholamine synthesis in the adrenal medulla, as well as increasing plasma catecholamine levels. [31]. Although it is understood that such networks can mediate coordination between organs in response to stress or dyshomeostasis in general, the specific impact of these interactions in the context of organ–organ crosstalk in ALD is incompletely understood.

### 3.3. Extracellular Vesicles

**Figure 2:**
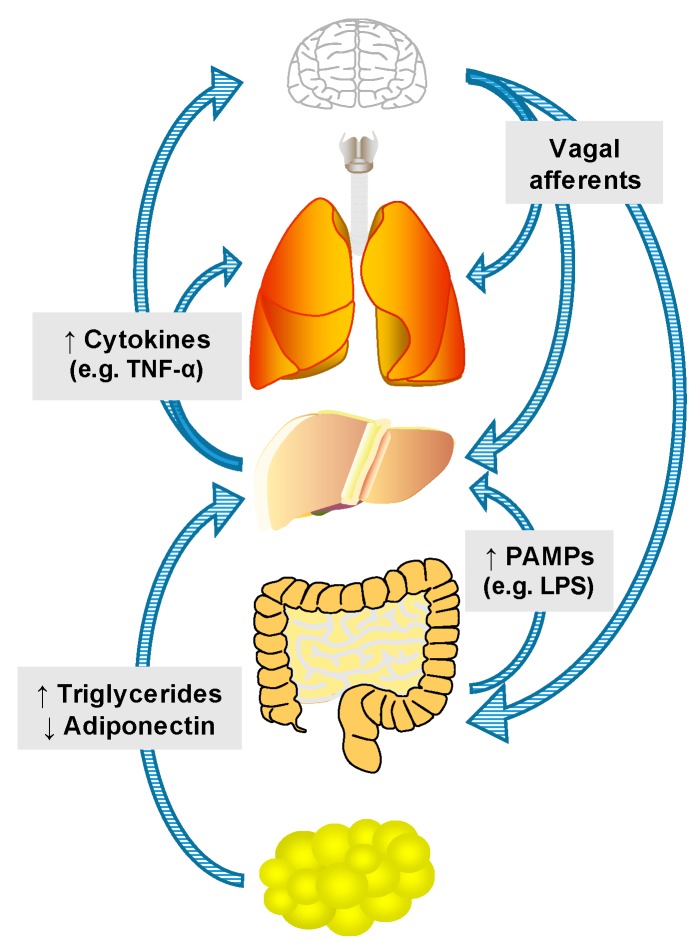
The liver at the center of organ–organ axes. There are several physiologic and pathophysiologic mechanisms by which inter-organ communication can be mediated, such as via nutrients, hormones and hepatokines (see Section 3.1), afferent and efferent innervation (Section 3.2), release of extracellular vesicles (Section 3.3), cytokines (Section 3.4) and pathogen-associated molecular patterns (PAMPs) and damage-associated molecular patterns (DAMPs) (Section 3.5). LPS: lipopolysaccharide; TNF-α: tumor necrosis factor-α.

Extracellular vesicles (EVs), a term that includes microvesicles (MV), exosomes, and apoptotic bodies, are an emerging mechanism of organ–organ communication in many diseases, including ALD [32]. Extracellular vesicles are defined as membrane-bound vesicles, ranging 0.1–1.0 μm in diameter, which are released from cells by budding of the cellular plasma membrane [32,33] (Figure 2). Extracellular vesicles can carry a diverse cargo, including lipids, proteins, RNAs, and miRNAs. One of the first studies to address the role of EVs in liver disease suggested that platelet-derived EVs could be used as a marker of hepatic fibrosis in chronic hepatitis C [34]. EVs have subsequently been implicated across the spectrum of liver diseases, including hepatitis viruses B and C, hepatocellular carcinoma, acute-on-chronic liver failure, non-alcoholic fatty liver disease (NAFLD) and ALD [32,35].

Extracellular vesicles are attractive therapeutic targets, due to both their potential mechanistic role in disease progression, as well as for being potential surrogate biomarkers. Over 10 years ago, it was proposed that MVs are (at least) surrogate biomarkers of advanced ALD [36]. Furthermore, a recent study described that alcohol exposure causes a release of hepatic EVs that contain a distinct miRNA profile [37]. It was also found that patients with ALD had elevated circulating EVs, and that these EVs also may carry a unique miRNA “barcode” [37]. EVs may not only serve as surrogate biomarkers of ALD, but they may also play a mechanistic role in disease progression. For example, ethanol-exposed HepG2 cells release EVs that subsequently activate cultured macrophages and primary Kupffer cells to the proinflammatory M1 phenotype [38]. CD40 ligand (CD40L), one of the EV proteins found in this in vitro study, was also found in EVs from sera of ALD patients. Importantly, genetic or pharmacologic, inhibition of CD40L protected against experimental ALD in mice, supporting the hypothesis that this EV cargo may have a mechanistic role in mediating disease [38].

In addition to mediating intra-organ communication between cells, EVs can also mediate inter-organ communication [39]. For example, adipose-derived EVs from genetically obese mice activate circulating monocytes when injected into lean mice [40]. Furthermore, remote preconditioning, such as the cardioprotective effect of hind limb ischemia-reperfusion injury, is also hypothesized to be mediated by EVs (e.g., [41,42]). The potential role of circulating EVs in mediating organ–organ communication in the context of ALD are not currently understood and would be an interesting target for further investigation.

### 3.4. Cytokines and Chemokines

Cytokines are a diverse group of small signaling molecules that mediate the immune and inflammatory responses. This group includes growth factors, interleukins, interferons, and chemokines, among others (Figure 2). A wide variety of cell types both produce and respond to cytokines, including inflammatory cells, such as monocytes, macrophages, and neutrophils, as well as parenchymal cells. Cytokines can be primarily classified into two groups: T helper 1 (Th1) and T helper 2 (Th2). The Th1 cytokines are typically considered “pro-inflammatory”, including tumor necrosis factor-α (TNF-α), interleukin (IL)-1, IL-6, and interferon. The Th2 cytokines are considered “anti-inflammatory” or “pro-fibrotic” and include IL-10 and IL-4. Homeostasis mediated by these cytokines ensures appropriate immune and inflammatory responses, with minimal normal tissue damage. These mediators also facilitate a coordinated response to insults and injuries that stem well beyond the primary target organ.

Dysregulated cytokine production has been implicated in the development and progression of liver diseases, including NAFLD and ALD [43]. Kupffer cells, the resident hepatic macrophages, play a key role in monitoring, clearing and mediating responses to gut-derived toxins, such as bacterial lipopolysaccharide (LPS). Alcohol consumption has long been known to increase gut permeability, leading to increased LPS in the portal circulation [44,45]. LPS and other bacterial toxins interact with Kupffer cells via toll-like receptors (TLRs) including TLR4. Activation of the TLR4 signaling pathway leads to increased transcription of several pro-inflammatory cytokines. It is therefore not surprising that patients with ALD have increased levels of several circulating cytokines, including TNF-α [46]. Other cytokines also likely play key roles, but TNF-α is likely the most well studied.

TNF-α is widely recognized as having a potential role in organ–organ communication in alcohol-induced organ injury. TNF-α is suspected to be a common mechanism of alcohol-induced pathology not only in the liver [47,48], but also in other organs, such as the lung and the brain. In the lung, chronic alcohol pre-exposure enhanced endotoxemia-induced acute lung injury (ALI), which was prevented by blocking systemic TNF-α with etanercept [49]. In the brain, alcohol exposure enhances the increase in TNF-α levels in caused by LPS, which is also prevented by deleting the canonical TNF-α receptor (TNFR1; [50]). However, experimental models also suggest that systemic TNF-α contributes to injury in organs that are not primary targets of alcohol abuse, including the lung and the brain. The source of this systemic TNF-α ultimately remains unknown, although the liver is clearly a likely key player. The potential for a liver–lung and liver–brain axis in the setting of chronic alcohol exposure will be discussed in a later section of this review.

### 3.5. PAMPs and DAMPs

As mentioned above, the gut-derived toxin, LPS, has long been identified as playing a potential role in ALD. The primary receptor for LPS (TLR4) belongs to a family of pattern recognition receptors (PRRs). Ligands for these receptors are grouped together as molecular “danger signals,” and include PAMPs and DAMPs (Figure 2). PAMPs include products of microbial (e.g., bacterial endotoxin or bacterial DNA), viruses, fungi, and parasites. DAMPs, on the other hand, are endogenous danger signals released from dead or dying cells; examples include extracellular DNA or RNA, free fatty acids, and high motility group box-1 (HMGB1), among others [51]. Signals derived by PAMPs/DAMPs can span multiple organs, and are thought to contribute to systemic inflammatory responses (e.g., SIRS; [52]). The innate immune system surveils qualitative and quantitative changes in the spectrum of PAMPs and DAMPs as a means to mount rapid and coordinated responses to any perceived threat that is driving those changes. Although this response is important for normal immune/inflammatory function, dysregulation of this response can lead to inappropriate inflammation and tissue damage. Ethanol consumption appears to broadly dysregulate the response of these receptors and enhances expression of several TLRs, as well as their responses to stimuli [53].

The involvement of PAMPs in organ–organ crosstalk is perhaps best understood in the context of the gut–liver axis. Intestinally-derived “toxins” were found to be normally present in the portal blood by Pavlov as early as 1893 [54]. Several years later, several studies indicated that experimental fibrosis induced by choline deficiency depended on intestinal-derived bacterial products. Rutenburg et al. demonstrated that non-absorbable antibiotics attenuated diet-induced fibrosis in rats [55]. The role of systemic PAMPs (e.g., LPS) in liver diseases is not limited to diet-induced cirrhosis, but is also established in models of toxicant-induced fibrosis (e.g., carbon tetrachloride (CCl_4_) administration), as well as other acute and chronic liver diseases [56,57]. High systemic levels of the PAMP, LPS, are found in both acute and chronic liver diseases [58,59].

Aside from the liver, GI-derived PAMPs during alcohol exposure can affect other organs, such as the brain. Although LPS cannot directly cross the blood–brain barrier, LPS injection increases brain inflammation. This increased inflammation in the brain is likely due to the increase in systemic inflammatory cytokines, such as TNF-α [50]. Interestingly, modulation of the gut barrier with nutritional therapies has yielded protective effects in human and animal studies [60,61,62]. The role of the gut permeability in the gut-liver axis in ALD will be discussed in detail in a later section of this review.

One of the best-understood DAMPs, or “alarmins”, is HMGB1. HMGB1 is a constitutively-expressed nuclear protein that is released from necrotic cells. A recent study demonstrated that HMGB1 translocation from the nucleus to the cytosol correlated with disease severity in liver biopsies from ALD patients [63]. Additionally, knocking out HMGB1 in hepatocytes protects mice against alcohol-induced liver injury. HMGB1 signaling has also been shown to be elevated in alcohol-induced injury in the brain [64,65] and pancreas [66]. However, the role of HMGB1 and other DAMPs in organ–organ crosstalk in the setting of ALD has been largely unexplored.

Although less characterized in the setting of ALD, the concept that circulating DAMPs may be involved in organ–organ crosstalk in other disease states is clearly established. For example, trauma elevates circulating mitochondrial DAMPs (MTDs), such as mitochondrial DNA, and is hypothesized to contribute to SIRS under that condition [67]. Human neutrophils exposed to MTDs have increased expression of the chemokine IL-8. In the same study, it was demonstrated that MTDs derived from rat liver cause lung injury in recipient rats; this injury was characterized by vascular leak, pulmonary edema, neutrophil infiltration, and accumulation of inflammatory cytokines in the alveolar space. As mentioned above, SIRS has been established as a significant risk factor for mortality from AH [19]. Therefore, it is not unlikely that circulating DAMPs may be involved. This would be an interesting target for further investigation.

## 4. Known Organ–Organ Interactions in Alcoholic Liver Disease

**Figure 3:**
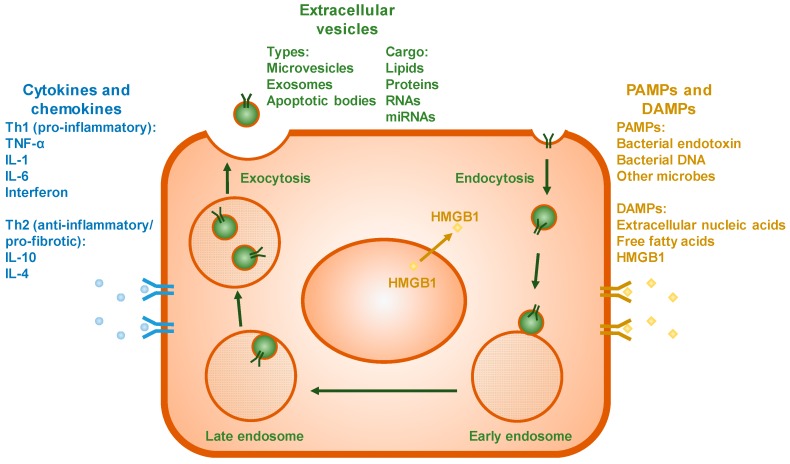
Specific mechanisms of communication in response to stress. Injured cells can release several signals to mediate communication between them and distal tissues. The profiles of both T helper 1 (Th1) (i.e., proinflammatory) and T helper 2 (Th2) (i.e., anti-inflammatory or profibrotic) cytokines is altered during hepatic injury. Additionally, the liver can respond to PAMPs (e.g., bacterial LPS) from other organs, such as the gastrointestinal (GI) tract, and/or respond or release DAMPs (e.g., high motility group box-1 (HMGB1)) that serve as ‘danger signals’ to pattern recognition receptors in other organs. IL: interleukin.

As mentioned previously, end-stage ALD is well-recognized as a systemic disorder. Communication between the liver and other organs has also been established, however, in earlier stages of the disease. This review will discuss potential interactions between the liver and the gut, the brain, adipose tissue, the lung, and the kidney (Figure 3).

### 4.1. Gut–Liver Interactions

As discussed in Section 3.4, the liver and the gut have established interactions in ALD (Figure 3). Specifically, alcohol feeding impairs gut barrier integrity, allowing for the leakage of bacterial products, including bacterial endotoxin, into portal circulation. The liver is therefore the first tissue to contact these gut-derived toxins. Furthermore, endotoxin (i.e., LPS) has a mechanistic role in alcohol-induced hepatic inflammation, as discussed previously in Section 3.3. Alcohol exposure is known to decrease expression of tight junction proteins in vivo [68] and in vitro [69]. Recently, mechanistic roles of miRNAs for control of gut tight junction proteins have been elucidated. For example, knockdown of miRNA-212 prevented ethanol-induced disruption of the tight junction protein, zonula occludens-1 (ZO-1), in the intestinal epithelium of mice exposed to chronic alcohol [70]. Additionally, inhibition of miRNA-122a protected against ethanol-induced occludin loss in Caco-2 cells [68]. In both of these cases, protection against alcohol-induced gut barrier dysfunction was associated with attenuation of alcohol-induced liver injury. 

One key aspect of gut-liver communication in ALD is alcohol-related gut dysbiosis. This topic has been recently reviewed extensively [71,72]. Using next generation sequencing, recent studies have described in detail the qualitative and quantitative changes in the gut microbiome in mice [73] and humans [74]. Interestingly, clinical studies have revealed that although not all patients who heavily consume alcohol develop gut dysbiosis, those that develop this condition have a higher degree of intestinal permeability than those who do not [75]. Furthermore, recent studies suggest that Diagnostic and Statistical Manual (DSM-V) mental disorders, including alcohol use disorders (AUDs), may have an underlying dysbiotic cause [76]. Taken together, these data have made the gut microbiome an attractive target for therapy in patients who abuse alcohol [77]. 

Probiotics (agents containing live bacterial cultures), prebiotics (agents that promote the growth of beneficial or commensal bacteria), and antibiotics have all been investigated as potential therapies for alcoholic liver disease. Recently, administration of various probiotic compounds was shown to protect against alcohol-induced liver injury in mice [78]. Clinically, administration of probiotics improved gut-dysbiosis and plasma transaminase levels in ALD patients [60]. Evidence suggests that factors secreted by commensal bacteria are themselves protective against alcohol-induced gut permeability and ALD. Indeed, the supernatant of *Lactobacillus rhamnosus* GG culture has been protective in mouse models of acute alcohol exposure [79], chronic alcohol exposure [80], and chronic plus binge alcohol exposure [81]. Prebiotics have also been demonstrated to attenuate alcohol-induced liver injury in animal models. Supplementation of an ethanol-containing diet with oats protected against alcohol-induced hepatic oxidative stress [82], and in another study, supplementation of a chronic alcohol-containing liquid diet with flaxseed oil protected against alcohol-induced hepatic inflammation and restored alcohol-induced gut dysbiosis [83]. Treatment with antibiotics has also been clinically demonstrated to attenuate alcohol-induced endotoxemia by preventing the overgrowth of harmful bacteria in the gut [84]. Indeed, the relationship of the gut microbiome and the liver in the setting of alcohol exposure is complicated. Germ-free mice showed enhanced susceptibility to acute alcohol toxicity by showing increased hepatic steatosis and an increased rate of ethanol metabolism [85]. Communication from the gut to the liver is an interesting target for further investigation.

This communication may also be bi-directional [72]. Chronic alcohol consumption alters the composition of bile produced in the liver. Alcohol exposure increases production of bile acids from the liver [86]. Bile acids prevent the growth of bacteria and may exert antimicrobial effects in the small intestine by binding to the bile acid receptor, farnesoid X receptor (FXR) [87]. Antimicrobial immunoglobulin A (IgA) also increases in livers of alcohol-exposed animals [88]. It is hypothesized that increases in hepatic-derived IgA may be a natural defense against alcohol-induced translocation of microbes and bacterial products [72]. Although the interactions between the gut and liver have been investigated extensively, further mechanistic insight would be beneficial for the development of effective therapeutics.

### 4.2. Liver–Adipose Interactions

Although patients with end-stage liver disease often exhibit significant muscle wasting, chronic alcohol consumption is often associated with being overweight. In fact, individuals who are overweight and heavily consume alcohol are at the most significant risk for developing serious fatty liver disease [89,90,91,92]. The liver–adipose axis in chronic alcohol exposure is relatively well-characterized, and has been extensively reviewed elsewhere [93]. For example, it is well-known that alcoholic patients exhibit a decrease in adipose fat, but an increase in liver fat [94]. Furthermore, blocking alcohol-induced adipose triglyceride depletion using dietary zinc supplements attenuates the development of alcoholic hepatic steatosis in mice [95]. However, the mechanism of “reverse triglyceride transport” has only recently been established experimentally [96,97]. Mice were exposed to deuterium water to label adipose triglycerides, and then fed an alcohol-containing liquid diet for two weeks. Alcohol feeding caused accumulation of deuterium-labelled triglycerides in the liver, thereby establishing that adipose-derived triglycerides contribute directly to alcoholic hepatic steatosis (Figure 3).

Adipose-derived signaling molecules, or adipokines, also contribute to the development of ALD. Adiponectin is an anti-inflammatory adipokine that has been shown to be decreased in the plasma of alcohol-fed rodents [98]. One potential mechanism by which alcohol blocks adiponectin release from adipose tissue is activation of the inflammatory response. Alcohol administration in rodents causes increased TNF-α expression in adipose tissue [99]. TNF-α directly inhibits the release of adiponectin from the adipose tissue [100], ultimately impairing lipid metabolism in target organs, such as the liver. Furthermore, administration of adiponectin protects against alcohol-induced hepatic steatosis and liver injury in mice [101]. The adipose-liver axis is an interesting target for further investigation.

### 4.3. Liver–Brain Interactions

Hepatic encephalopathy, or declining brain functions in patients with liver disease, is one manifestation of decompensated ALD. However, evidence suggests that interactions between the liver and brain, as well as the gut, may contribute to alcohol-induced brain injury and inflammation even with mild alcohol consumption (Figure 3). For example, the brain is responsive to systemic inflammation. Cytokines in the brain, such as TNF-α, are elevated after systemic LPS injection, even though LPS itself does not cross the blood–brain barrier [102]. Furthermore, as mentioned previously, mice lacking TNFR1 demonstrated elevated TNF-α in liver and serum, but not in the brain [103]. Similarly, mice exposed to intragastric ethanol display elevated TNF-α in the brain [102].

Not only is the brain susceptible to alcohol-induced systemic inflammation, but the brain itself may also contribute to this pathology. For example, alcohol exposure interferes with the stress response via disruption of the hypothalamo–pituitary–adrenal (HPA) axis (reviewed in [104]). In animal studies involving self-administration of ethanol, “non-dependent” animals showed elevated blood cortisol levels after alcohol consumption. On the other hand, “dependent” animals, who voluntarily consumed higher amounts of alcohol, showed blunted blood cortisol levels, suggesting that chronic alcohol abuse produces tolerance to the alcohol-induced stress response [105]. Therefore, alcohol-induced suppression of the HPA axis may be a potential mechanism by which systemic inflammation persists in individuals who chronically consume alcohol.

Finally, communication between the gut, liver, and brain is also an important aspect of organ–organ crosstalk during alcohol exposure. As previously discussed, the loss of gut barrier integrity after alcohol exposure is a primary cause of endotoxemia in alcoholic patients. Gut-derived LPS activates hepatic macrophages to produce TNF-α, which, along with other systemic sources of this cytokine, may contribute to inflammation in the brain. Furthermore, alcohol-mediated changes in the gut, including gut dysbiosis, may also contribute to alcohol dependence-related behaviors [106]. Indeed, the gut microbiome is thought to play a role in controlling different aspects of behavior, potentially through endocrine signaling, neural signaling, and the immune system [107]. Furthermore, one study shows that treating patients with depression, who also display systemic inflammation, with an inhibitor of TNF-α showed improved symptoms [108]. However, a direct link between alcohol-induced dysbiosis and alcohol dependence behavior is yet to be established, and is an interesting target for further investigation.

### 4.4. Liver–Lung Interactions

The idea of the liver–lung axis in the setting of chronic alcohol exposure is based on clinical data demonstrating that patients with a diagnosed alcohol use disorder have increased susceptibility to bacterial infection, increased incidence of and mortality from acute respiratory distress syndrome (ARDS) and causes hepatopulmonary syndrome [109,110,111]. Furthermore, in ARDS patients with hepatic failure, mortality increases to almost 100% [110]. Pulmonary injury induced by LPS can be altered by mediators released from the liver (e.g., TNF-α). Indeed, in an elegant study by Siore et al., LPS-induced lung damage required perfusion with the liver [112].

**Figure 4:**
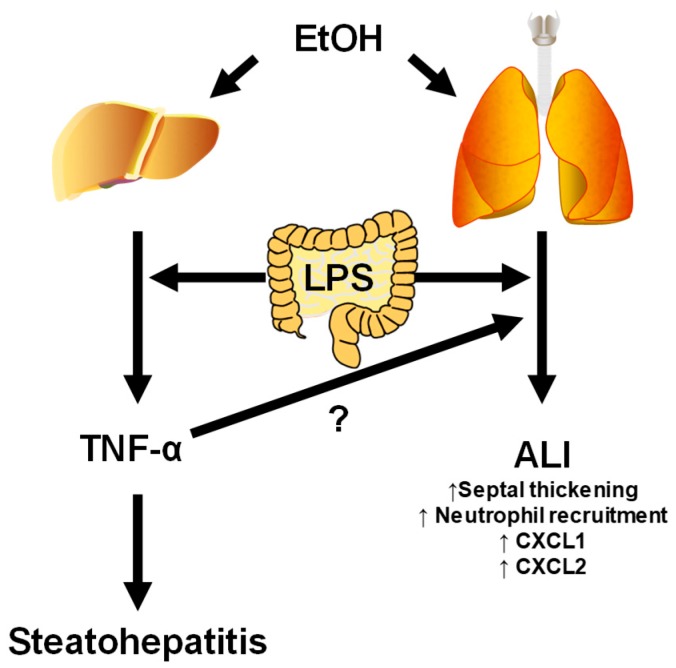
Hypothesized axis between the liver and lung in ALI. Ethanol pre-exposure primes both the liver and lung to enhanced injury and inflammation caused by PAMPs (e.g., LPS). Furthermore, the release of proinflammatory cytokines (e.g., TNF-α) into the systemic circulation from the liver may enhance inflammatory lung injury in the context of ALI. CXCL: C-X-C motif ligand.

In a more recent study, mice were exposed to chronic alcohol on the Lieber DeCarli liquid diet for six weeks, followed by intraperitoneal injection of LPS [49]. The differential effects on cytokine expression in systemic circulation and locally in the lung (i.e., bronchoalveolar lavage fluid (BALF)) were examined. Animals pre-exposed to an ethanol diet had significantly elevated levels of plasma TNF-α after LPS injection compared to animals fed a control diet. In the BALF, however, ethanol pre-exposed animals had elevated levels of the TNF-α-responsive chemokines, macrophage inflammatory protein (MIP)-2 and keratinocyte chemoattractant (KC). This elevated chemokine expression also correlated with increased pulmonary neutrophil recruitment. Interestingly, blocking systemic TNF-α using a TNF-α-inhibiting antibody, etanercept, significantly attenuated the alcohol-enhanced pulmonary chemokine expression, and ultimately, alcohol-enhanced lung injury and inflammation after LPS (Figure 4). While the liver is not the sole source of systemic TNF-α in this experimental setting, other studies have demonstrated that ablation of Kupffer cells before LPS injection decreased systemic TNF-α levels by almost 90%, indicating that these cells are in fact a predominate source of plasma TNF-α in experimental endotoxemia [113]. Future studies should aim to develop a more specific system to directly examine the role of hepatic-derived cytokines in alcohol-enhanced pulmonary injury. As in the other axes of injury discussed in this review, communication between the liver and lung during injury may also be bi-directional. In a mouse model of ventilator-induced lung injury, perfusate from injured lungs caused a robust inflammatory response in cultured hepatic sinusoidal endothelial cells [114], suggesting lung-derived mediators may contribute to liver inflammation in this disease model. However, there are currently no studies investigating the role of lung-derived mediators in alcohol-induced liver injury; this would be an interesting target for further investigation.

## 5. Summary and Conclusions

In conclusion, while multiple organ failure is a hallmark of decompensated, end-stage alcoholic liver disease, there is an increasing appreciation for communication between organs during earlier stages of the disease. Organs can communicate with one another via several potential mechanisms, including extracellular vesicles, cytokines and chemokines, PAMPs and DAMPs, and the nervous system (Figure 2). The liver is proposed to communicate with other organs, such as the gut, brain, lung, and adipose tissue using these mechanisms, as well as others (e.g., muscle; Figure 3). Understanding the mechanisms by which organs communicate during the inflammatory injury phase of ALD may allow for the development of targeted therapeutics to protect one or all of these systems from alcohol-mediated toxicities.

## References

[1] Substance Abuse and Mental Health Services Administration (2015). 2015 National Survey on Drug Use and Health.

[2] World Health Organization (2014). Global Status Report on Alcohol and Health.

[3] Beier J.I., Arteel G.E., McClain C.J. (2011). Advances in Alcoholic Liver Disease. Curr. Gastroenterol. Rep..

[4] Guidot D.M., Roman J. (2002). Chronic Ethanol Ingestion Increases Susceptibility to Acute Lung Injury: Role of Oxidative Stress and Tissue Remodeling. Chest.

[5] Adachi J., Asano M., Ueno Y., Niemela O., Ohlendieck K., Peters T.J., Preedy V.R. (2003). Alcoholic Muscle Disease and Biomembrane Perturbations (Review). J. Nutr. Biochem..

[6] Crews F.T., Nixon K. (2009). Mechanisms of Neurodegeneration and Regeneration in Alcoholism. Alcohol Alcohol..

[7] Pandol S.J., Raraty M. (2007). Pathobiology of Alcoholic Pancreatitis. Pancreatology.

[8] IARC Working Group on the Evaluation of Carcinogenic Risks to Humans (2010). Alcohol Consumption and Ethyl Carbamate. IARC Monogr. Eval. Carcinog. Risks Hum..

[9] U.S. Burden of Disease Collaborators (2013). The State of U.S. Health, 1990–2010: Burden of Diseases, Injuries, and Risk Factors. JAMA.

[10] Levitt M.D., Levitt D.G., Furne J., DeMaster E.G. (1994). Can the Liver Account for First-Pass Metabolism of Ethanol in the Rat?. Am. J. Physiol..

[11] Lelbach W.K. (1966). Liver Damage in Chronic Alcoholism: Results of a Clinical, Clinical-Chemical and Bioptic-Histological Study in 526 Alcoholic Patients During a Low Calorie Diet in an Open Drinking Sanatorium. Acta Hepatosplenol..

[12] Mann R.E., Smart R.G., Govoni R. (2003). The Epidemiology of Alcoholic Liver Disease. Alcohol Res. Health.

[13] Day C.P. (2000). Who Gets Alcoholic Liver Disease: Nature or Nurture?. J. R. Coll. Physicians Lond..

[14] Diehl A.M. (2002). Liver Disease in Alcohol Abusers: Clinical Perspective. Alcohol.

[15] Powell W.J., Klatskin G. (1968). Duration of Survival in Patients with Laennec’s Cirrhosis. Influence of Alcohol Withdrawal, and Possible Effects of Recent Changes in General Management of the Disease. Am. J. Med..

[16] La Vecchia C., Negri E., D’Avanzo B., Boyle P., Franceschi S. (1990). Medical History and Primary Liver Cancer. Cancer Res..

[17] Gogel B.M., Goldstein R.M., Kuhn J.A., McCarty T.M., Donahoe A., Glastad K. (2000). Diagnostic Evaluation of Hepatocellular Carcinoma in a Cirrhotic Liver. Oncology.

[18] Moreau R., Jalan R., Gines P., Pavesi M., Angeli P., Cordoba J., Durand F., Gustot T., Saliba F., Domenicali M. (2013). Acute-on-Chronic Liver Failure Is a Distinct Syndrome That Develops in Patients with Acute Decompensation of Cirrhosis. Gastroenterology.

[19] Michelena J., Altamirano J., Abraldes J.G., Affò S., Morales-Ibanez O., Sancho-Bru P., Dominguez M., García-Pagán J.C., Fernández J., Arroyo V. (2015). Systemic Inflammatory Response and Serum Lipopolysaccharide Levels Predict Multiple Organ Failure and Death in Alcoholic Hepatitis. Hepatology.

[20] Rui L. (2014). Energy Metabolism in the Liver. Compr. Physiol..

[21] Iroz A., Couty J.P., Postic C. (2015). Hepatokines: Unlocking the Multi-Organ Network in Metabolic Diseases. Diabetologia.

[22] Oh K.J., Lee D.S., Kim W.K., Han B.S., Lee S.C., Bae K.H. (2016). Metabolic Adaptation in Obesity and Type II Diabetes: Myokines, Adipokines and Hepatokines. Int. J. Mol. Sci..

[23] Lebensztejn D.M., Flisiak-Jackiewicz M., Bialokoz-Kalinowska I., Bobrus-Chociej A., Kowalska I. (2016). Hepatokines and Non-Alcoholic Fatty Liver Disease. Acta Biochim. Pol..

[24] Yamada T., Oka Y., Katagiri H. (2008). Inter-Organ Metabolic Communication Involved in Energy Homeostasis: Potential Therapeutic Targets for Obesity and Metabolic Syndrome. Pharmacol. Ther..

[25] Ferro J.M., Oliveira S. (2014). Neurologic Manifestations of Gastrointestinal and Liver Diseases. Curr. Neurol. Neurosci. Rep..

[26] Anthony D.C., Couch Y. (2014). The Systemic Response to CNS Injury. Exp. Neurol..

[27] De la Monte S.M. (2017). Insulin Resistance and Neurodegeneration: Progress towards the Development of New Therapeutics for Alzheimer’s Disease. Drugs.

[28] Kingman G.I., Goodall M. (1957). Urinary Epinephrine and Levarterenol Excretion During Acute Sublethal Alcohol Intoxication in Dogs. J. Pharmacol. Exp. Ther..

[29] Bravo I.R., Acevedo C.G., Gallardo V. (1980). Acute Effects of Ethanol on Liver Blood Circulation in the Anesthetized Dog. Alcohol. Clin. Exp. Res..

[30] Yuki T., Bradford B.U., Thurman R.G. (1980). Role of Hormones in the Mechanism of the Swift Increase in Alcohol Metabolism in the Rat. Pharmacol. Biochem. Behav..

[31] Patterson-Buckendahl P., Kubovcakova L., Krizanova O., Pohorecky L.A., Kvetnansky R. (2005). Ethanol Consumption Increases Rat Stress Hormones and Adrenomedullary Gene Expression. Alcohol.

[32] Maji S., Matsuda A., Yan I.K., Parasramka M., Patel T. (2017). Extracellular Vesicles in Liver Diseases. Am. J. Physiol. Gastrointest. Liver Physiol..

[33] Buzas E.I., Gyorgy B., Nagy G., Falus A., Gay S. (2014). Emerging Role of Extracellular Vesicles in Inflammatory Diseases. Nat. Rev. Rheumatol..

[34] Fusegawa H., Shiraishi K., Ogasawara F., Shimizu M., Haruki Y., Miyachi H., Matsuzaki S., Ando Y. (2002). Platelet Activation in Patients with Chronic Hepatitis C. Tokai J. Exp. Clin. Med..

[35] Hirsova P., Ibrahim S.H., Verma V.K., Morton L.A., Shah V.H., LaRusso N.F., Gores G.J., Malhi H. (2016). Extracellular Vesicles in Liver Pathobiology: Small Particles with Big Impact. Hepatology.

[36] Ogasawara F.F. (2005). Platelet Activation in Patients with Alcoholic Liver Disease. Tokai J. Exp. Clin. Med..

[37] Eguchi A., Lazaro R.G., Wang J., Kim J., Povero D., Willliams B., Ho S.B., Stärkel P., Schnabl B., Ohno-Machado L. (2017). Extracellular Vesicles Released by Hepatocytes from Gastric Infusion Model of Alcoholic Liver Disease Contain a MicroRNA Barcode That Can Be Detected in Blood. Hepatology.

[38] Verma V.K., Li H., Wang R., Hirsova P., Mushref M., Liu Y., Cao S., Contreras P.C., Malhi H., Kamath P.S. (2016). Alcohol Stimulates Macrophage Activation through Caspase Dependent Hepatocyte Derived Release of CD40L Containing Extracellular Vesicles. J. Hepatol..

[39] Yoshioka Y., Katsuda T., Ochiya T. (2015). Circulating MicroRNAs as Hormones: Intercellular and Inter-Organ Conveyors of Epigenetic Information?. EXS..

[40] Deng Z.B., Poliakov A., Hardy R.W., Clements R., Liu C., Liu Y., Wang J., Xiang X., Zhang S., Zhuang X. (2009). Adipose Tissue Exosome-Like Vesicles Mediate Activation of Macrophage-Induced Insulin Resistance. Diabetes.

[41] Ma F., Liu H., Shen Y., Zhang Y., Pan S. (2015). Platelet-Derived Microvesicles Are Involved in Cardio-Protective Effects of Remote Preconditioning. Int. J. Clin. Exp. Pathol..

[42] Giricz Z., Varga Z.V., Baranyai T., Sipos P., Paloczi K., Kittel A., Buzas E.I., Ferdinandy P. (2014). Cardioprotection by Remote Ischemic Preconditioning of the Rat Heart Is Mediated by Extracellular Vesicles. J. Mol. Cell. Cardiol..

[43] McClain C.J., Barve S., Deaciuc I., Kugelmas M., Hill D. (1999). Cytokines in Alcoholic Liver Disease. Semin. Liver Dis..

[44] Bode C.H., Kugler V., Bode J.C.H. (1987). Endotoxemia in Patients with Alcoholic and Non-Alcoholic Cirrhosis and in Subjects with No Evidence of Chronic Liver Disease Following Acute Alcohol Excess. J. Hepatol..

[45] Nolan J.P. (1975). The Role of Endotoxin in Liver Injury. Gastroenterology.

[46] Khoruts A., Stahnke L., McClain C.J., Logan G., Allen J.I. (1991). Circulating Tumor Necrosis Factor, Interleukin-1 and Interleukin-6 Concentrations in Chronic Alcoholic Patients. Hepatology.

[47] Iimuro Y., Gallucci R.M., Luster M.I., Kono H., Thurman R.G. (1997). Antibodies to Tumor Necrosis Factor-α Attenuate Hepatic Necrosis and Inflammation Due to Chronic Exposure to Ethanol in the Rat. Hepatology.

[48] Yin M., Wheeler M.D., Kono H., Bradford B.U., Gallucci R.M., Luster M.I., Thurman R.G. (1999). Essential Role of Tumor Necrosis Factor α in Alcohol-Induced Liver Injury. Gastroenterology.

[49] Massey V.L., Poole L.G., Siow D.L., Torres E., Warner N.L., Schmidt R.H., Ritzenthaler J.D., Roman J., Arteel G.E. (2015). Chronic Alcohol Exposure Enhances Lipopolysaccharide-Induced Lung Injury in Mice: Potential Role of Systemic Tumor Necrosis Factor-α. Alcohol. Clin. Exp. Res..

[50] Qin L., Wu X., Block M.L., Liu Y., Breese G.R., Hong J.S., Knapp D.J., Crews F.T. (2007). Systemic LPS Causes Chronic Neuroinflammation and Progressive Neurodegeneration. GLIA.

[51] Tilg H., Moschen A.R., Szabo G. (2016). Interleukin-1 and Inflammasomes in Alcoholic Liver Disease/Acute Alcoholic Hepatitis and Nonalcoholic Fatty Liver Disease/Nonalcoholic Steatohepatitis. Hepatology.

[52] Hirsiger S., Simmen H.P., Werner C.M., Wanner G.A., Rittirsch D. (2012). Danger Signals Activating the Immune Response After Trauma. Mediat. Inflamm..

[53] Gustot T., Lemmers A., Moreno C., Nagy N., Quertinmont E., Nicaise C., Franchimont D., Louis H., Deviere J., Le M.O. (2006). Differential Liver Sensitization to Toll-Like Receptor Pathways in Mice with Alcoholic Fatty Liver. Hepatology.

[54] Pavlov M. (1893). The Anti-Toxic Function of the Liver. Lancet.

[55] Rutenburg A.M., Sonnenblick E., Koven I., Aprahamian H.A., Reiner L., Fine J. (1957). The Role of Intestinal Bacteria in the Development of Dietary Cirrhosis in Rats. J. Exp. Med..

[56] Nolan J.P., Ali M.V. (1973). Endotoxin and the Liver. II Effect of Tolerance on Carbon Tetrachloride Induced Injury. J. Med..

[57] Nolan J.P., Leibowitz A.I. (1978). Endotoxin and the Liver. III. Modification of Acute Carbon Tetrachloride Injury by Polymyxin B—An Antiendotoxin. Gastroenterology.

[58] Wilkinson S.P., Arroyo V., Gazzard B.G., Moodie H., Williams R. (1974). Relation of Renal Impairment and Haemorrhagic Diathesis to Endotoxaemia in Fulminant Hepatic Failure. Lancet.

[59] Tarao K., Moroi T., Nagakura Y., Ikeuchi T., Suyama T., Endo O., Fukushima K. (1979). Relationship Between Endotoxaemia and Protein Concentration of Ascites in Cirrhotic Patients. Gut.

[60] Kirpich I.A., Solovieva N.V., Leikhter S.N., Shidakova N.A., Lebedeva O.V., Sidorov P.I., Bazhukova T.A., Soloviev A.G., Barve S.S., McClain C.J. (2008). Probiotics Restore Bowel Flora and Improve Liver Enzymes in Human Alcohol-Induced Liver Injury: A Pilot Study. Alcohol.

[61] Lambert J.C., Zhou Z., Wang L., Song Z., McClain C.J., Kang Y.J. (2003). Prevention of Alterations in Intestinal Permeability Is Involved in Zinc Inhibition of Acute Ethanol-Induced Liver Damage in Mice. J. Pharmacol. Exp. Ther..

[62] Segawa S., Wakita Y., Hirata H., Watari J. (2008). Oral Administration of Heat-Killed *Lactobacillus Brevis* SBC8803 Ameliorates Alcoholic Liver Disease in Ethanol-Containing Diet-Fed C57BL/6N Mice. Int. J. Food Microbiol..

[63] Ge X., Antoine D.J., Lu Y., Arriazu E., Leung T.M., Klepper A.L., Branch A.D., Fiel M.I., Nieto N. (2014). High Mobility Group Box-1 (HMGB1) Participates in the Pathogenesis of Alcoholic Liver Disease (ALD). J. Biol. Chem..

[64] Coleman L.G., Zou J., Crews F.T. (2017). Microglial-Derived miRNA let-7 and HMGB1 Contribute to Ethanol-Induced Neurotoxicity via TLR7. J. Neuroinflamm..

[65] Zou J.Y., Crews F.T. (2014). Release of Neuronal HMGB1 by Ethanol through Decreased HDAC Activity Activates Brain Neuroimmune Signaling. PLoS ONE.

[66] Ren Z., Wang X., Xu M., Yang F., Frank J.A., Ke Z., Luo J. (2016). Binge Ethanol Exposure Causes Endoplasmic Reticulum Stress, Oxidative Stress and Tissue Injury in the Pancreas. Oncotarget.

[67] Zhang Q., Raoof M., Chen Y., Sumi Y., Sursal T., Junger W., Brohi K., Itagaki K., Hauser C.J. (2010). Circulating Mitochondrial DAMPs Cause Inflammatory Responses to Injury. Nature.

[68] Zhao H., Zhao C., Dong Y., Zhang M., Wang Y., Li F., Li X., McClain C., Yang S., Feng W. (2015). Inhibition of miR122a by *Lactobacillus Rhamnosus* GG Culture Supernatant Increases Intestinal Occludin Expression and Protects Mice from Alcoholic Liver Disease. Toxicol. Lett..

[69] Tang Y., Zhang L., Forsyth C.B., Shaikh M., Song S., Keshavarzian A. (2015). The Role of miR-212 and iNOS in Alcohol-Induced Intestinal Barrier Dysfunction and Steatohepatitis. Alcohol. Clin. Exp. Res..

[70] Pettinelli P., Videla L.A. (2011). Up-Regulation of PPAR-γ mRNA Expression in the Liver of Obese Patients: An Additional Reinforcing Lipogenic Mechanism to SREBP-1c Induction. J. Clin. Endocrinol. Metab..

[71] Dasarathy S., Brown J.M. (2017). Alcoholic Liver Disease on the Rise: Interorgan Cross Talk Driving Liver Injury. Alcohol. Clin. Exp. Res..

[72] Stärkel P., Schnabl B. (2016). Bidirectional Communication between Liver and Gut during Alcoholic Liver Disease. Semin. Liver Dis..

[73] Bull-Otterson L., Feng W., Kirpich I., Wang Y., Qin X., Liu Y., Gobejishvili L., Joshi-Barve S., Ayvaz T., Petrosino J. (2013). Metagenomic Analyses of Alcohol Induced Pathogenic Alterations in the Intestinal Microbiome and the Effect of *Lactobacillus Rhamnosus* GG Treatment. PLoS ONE.

[74] Mutlu E.A., Gillevet P.M., Rangwala H., Sikaroodi M., Naqvi A., Engen P.A., Kwasny M., Lau C.K., Keshavarzian A. (2012). Colonic Microbiome Is Altered in Alcoholism. Am. J. Physiol. Gastrointest. Liver Physiol..

[75] Leclercq S., Matamoros S., Cani P.D., Neyrinck A.M., Jamar F., Stärkel P., Windey K., Tremaroli V., Bäckhed F., Verbeke K. (2014). Intestinal Permeability, Gut-Bacterial Dysbiosis, and Behavioral Markers of Alcohol-Dependence Severity. Proc. Natl. Acad. Sci. USA.

[76] Temko J.E., Bouhlal S., Farokhnia M., Lee M.R., Cryan J.F., Leggio L. (2017). The Microbiota, the Gut and the Brain in Eating and Alcohol Use Disorders: A ‘Menage a Trois’?. Alcohol Alcohol..

[77] Sung H., Kim S.W., Hong M., Suk K.T. (2016). Microbiota-Based Treatments in Alcoholic Liver Disease. World J. Gastroenterol..

[78] Tian F., Chi F., Wang G., Liu X., Zhang Q., Chen Y., Zhang H., Chen W. (2015). *Lactobacillus Rhamnosus* CCFM1107 Treatment Ameliorates Alcohol-Induced Liver Injury in a Mouse Model of Chronic Alcohol Feeding. J. Microbiol..

[79] Wang Y., Liu Y., Sidhu A., Ma Z., McClain C., Feng W. (2012). *Lactobacillus Rhamnosus* GG Culture Supernatant Ameliorates Acute Alcohol-Induced Intestinal Permeability and Liver Injury. Am. J. Physiol. Gastrointest. Liver Physiol..

[80] Wang Y., Liu Y., Kirpich I., Ma Z., Wang C., Zhang M., Suttles J., McClain C., Feng W. (2013). *Lactobacillus Rhamnosus* GG Reduces Hepatic TNFα Production and Inflammation in Chronic Alcohol-Induced Liver Injury. J. Nutr. Biochem..

[81] Chen R.C., Xu L.M., Du S.J., Huang S.S., Wu H., Dong J.J., Huang J.R., Wang X.D., Feng W.K., Chen Y.P. (2016). *Lactobacillus Rhamnosus* GG Supernatant Promotes Intestinal Barrier Function, Balances T_reg_ and T_H_17 Cells and Ameliorates Hepatic Injury in a Mouse Model of Chronic-Binge Alcohol Feeding. Toxicol. Lett..

[82] Tang Y., Forsyth C.B., Banan A., Fields J.Z., Keshavarzian A. (2009). Oats Supplementation Prevents Alcohol-Induced Gut Leakiness in Rats by Preventing Alcohol-Induced Oxidative Tissue Damage. J. Pharmacol. Exp. Ther..

[83] Zhang X., Wang H., Yin P., Fan H., Sun L., Liu Y. (2017). Flaxseed Oil Ameliorates Alcoholic Liver Disease via Anti-Inflammation and Modulating Gut Microbiota in Mice. Lipids Health Dis..

[84] Kalambokis G.N., Mouzaki A., Rodi M., Tsianos E.V. (2012). Rifaximin Improves Thrombocytopenia in Patients with Alcoholic Cirrhosis in Association with Reduction of Endotoxaemia. Liver Int..

[85] Chen P., Miyamoto Y., Mazagova M., Lee K.C., Eckmann L., Schnabl B. (2015). Microbiota Protects Mice Against Acute Alcohol-Induced Liver Injury. Alcohol. Clin. Exp. Res..

[86] Xie G., Zhong W., Li H. (2013). Alteration of Bile Acid Metabolism in the Rat Induced by Chronic Ethanol Consumption. FASEB J..

[87] Inagaki T., Moschetta A., Lee Y. (2006). Regulation of Antibacterial Defense in the Small Intestine by the Nuclear Bile Acid Receptor. Proc. Natl. Acad. Sci. USA.

[88] Moro-Sibilot L., Blanc P., Taillardet M. (2016). Mouse and Human Liver Contain Immunoglobulin a-Secreting Cells Originating from Peyer’s Patches and Directed Against Intestinal Antigens. Gastroenterology.

[89] Dunn W., Zeng Z., O’Neil M., Zhao J., Whitener M., Yu-Jui W.Y., Mitchell E.K., Handler M., Weinman S.A. (2012). The Interaction of Rs738409, Obesity, and Alcohol: A Population-Based Autopsy Study. Am. J. Gastroenterol..

[90] Ruhl C.E., Everhart J.E. (2005). Joint Effects of Body Weight and Alcohol on Elevated Serum Alanine Aminotransferase in the United States Population. Clin. Gastroenterol. Hepatol..

[91] Hart C.L., Morrison D.S., Batty G.D., Mitchell R.J., Davey S.G. (2010). Effect of Body Mass Index and Alcohol Consumption on Liver Disease: Analysis of Data from Two Prospective Cohort Studies. BMJ.

[92] Alatalo P.I., Koivisto H.M., Hietala J.P., Puukka K.S., Bloigu R., Niemela O.J. (2008). Effect of Moderate Alcohol Consumption on Liver Enzymes Increases with Increasing Body Mass Index. Am. J. Clin. Nutr..

[93] Steiner J.L., Lang C.H. (2017). Alcohol, Adipose Tissue, and Lipid Dysregulation. Biomolecules.

[94] Addolorato G., Capristo E., Greco A.V., Stefanini G.F., Gasbarrini G. (1997). Energy Expenditure, Substrate Oxidation, and Body Composition in Subjects with Chronic Alcoholism: New Findings from Metabolic Assessment. Alcohol. Clin. Exp. Res..

[95] Kang X., Zhong W., Liu J., Song Z., McClain C.J., Kang Y.J., Zhou Z. (2009). Zinc Supplementation Reverses Alcohol-Induced Steatosis in Mice Through Reactivating Hepatocyte Nuclear Factor-4α and Peroxisome Proliferators Activated Receptor-α. Hepatology.

[96] Zhong W., Zhao Y., Tang Y., Wei X., Shi X., Sun W., Sun X., Yin X., Sun X., Kim S. (2012). Chronic Alcohol Exposure Stimulates Adipose Tissue Lipolysis in Mice: Role of Reverse Triglyceride Transport in the Pathogenesis of Alcoholic Steatosis. Am. J. Pathol..

[97] Wei X., Shi X., Zhong W., Zhao Y., Tang Y., Sun W., Yin X., Bogdanov B., Kim S., McClain C. (2013). Chronic Alcohol Exposure Disturbs Lipid Homeostasis at the Adipose Tissue-Liver Axis in Mice: Analysis of Triacylglycerols Using High-Resolution Mass Spectrometry in Combination with in Vivo Metabolite Deuterium Labeling. PLoS ONE.

[98] Chen X., Sebastian B.M., Tang H., McMullen M.M., Axhemi A., Jacobsen D.W., Nagy L.E. (2009). Taurine Supplementation Prevents Ethanol-Induced Decrease in Serum Adiponectin and Reduces Hepatic Steatosis in Rats. Hepatology.

[99] He Z., Li M., Zheng D., Chen Q., Liu W., Feng L. (2015). Adipose Tissue Hypoxia and Low-Grade Inflammation: A Possible Mechanism for Ethanol-Related Glucose Intolerance?. Br. J. Nutr..

[100] Zappalà G., Rechler M.M. (2009). IGFBP-3, Hypoxia and TNF-α Inhibit Adiponectin Transcription. Biochem. Biophys. Res. Commun..

[101] Xu A., Wang Y., Keshaw H., Xu L.Y., Lam K.S., Cooper G.J. (2003). The Fat-Derived Hormone Adiponectin Alleviates Alcoholic and Nonalcoholic Fatty Liver Diseases in Mice. J. Clin. Investig..

[102] Qin L., He J., Hanes R.N., Pluzarev O., Hong J.S., Crews F.T. (2008). Increased Systemic and Brain Cytokine Production and Neuroinflammation by Endotoxin Following Ethanol Treatment. J. Neuroinflamm..

[103] Mayfield J., Ferguson L., Harris R.A. (2013). Neuroimmune Signaling: A Key Component of Alcohol Abuse. Curr. Opin. Neurobiol..

[104] Wang H.J., Zakhari S., Jung M.K. (2010). Alcohol, Inflammation, and Gut–Liver–Brain Interactions in Tissue Damage and Disease Development. World J. Gastroenterol..

[105] Richardson H.N., Lee S.Y., O’Dell L.E., Koob G.F., Rivier C.L. (2008). Alcohol Self-Administration Acutely Stimulates the Hypothalamic-Pituitary-Adrenal Axis, but Alcohol Dependence Leads to a Dampened Neuroendocrine State. Eur. J. Neurosci..

[106] Leclercq S., de Timary P., Delzenne N.M., Starkel P. (2017). The Link Between Inflammation, Bugs, the Intestine and the Brain in Alcohol Dependence. Transl. Psychiatry.

[107] Cryan J.F., Dinan T.G. (2012). Mind-Altering Microorganisms: The Impact of the Gut Microbiota on Brain and Behaviour. Nat. Rev. Neurosci..

[108] Raison C.L., Rutherford R.E., Woolwine B.J., Shuo C., Schettler P., Drake D.F., Haroon E., Miller A.H. (2013). A Randomized Controlled Trial of the Tumor Necrosis Factor-α Antagonist Infliximab in Treatment Resistant Depression: Role of Baseline Inflammatory Biomarkers. JAMA Psychiatry.

[109] Afshar M., Smith G.S., Terrin M.L., Barrett M., Lissauer M.E., Mansoor S., Jeudy J., Netzer G. (2014). Blood Alcohol Content, Injury Severity and Acute Respiratory Distress Syndrome. J. Trauma Acute Care Surg..

[110] Moss M., Bucher B., Moore F.A., Moore E.E., Parsons P.E. (1996). The Role of Chronic Alcohol Abuse in the Development of Acute Respiratory Distress Syndrome in Adults. JAMA.

[111] Moss M., Parsons P.E., Steinberg K.P., Hudson L.D., Guidot D.M., Burnham E.L., Eaton S., Cotsonis G.A. (2003). Chronic Alcohol Abuse Is Associated with an Increased Incidence of Acute Respiratory Distress Syndrome and Severity of Multiple Organ Dysfunction in Patients with Septic Shock. Crit. Care Med..

[112] Siore A.M., Parker R.E., Stecenko A.A., Cuppels C., McKean M., Christman B.W., Cruz-Gervis R., Brigham K.L. (2005). Endotoxin-Induced Acute Lung Injury Requires Interaction with the Liver. Am. J. Physiol. Lung Cell. Mol. Physiol..

[113] Bautista A.P., Skrepnik N., Niesman M.R., Bagby G.J. (1994). Elimination of Macrophages by Liposome-Encapsulated Dichlorovethylene Diphosphonate Suppresses the Endotoxin-Induced Priming of Kupffer Cells. J. Leukoc. Biol..

[114] Patterson E.K., Yao L.J., Ramic N., Lewis J.F., Cepinskas G., McCaig L., Veldhuizen R.A., Yamashita C.M. (2013). Lung-Derived Mediators Induce Cytokine Production in Downstream Organs via an NF-κB-Dependent Mechanism. Mediat. Inflamm..

